# CD137 Agonists Targeting CD137-Mediated Negative Regulation Show Enhanced Antitumor Efficacy in Lung Cancer

**DOI:** 10.3389/fimmu.2022.771809

**Published:** 2022-02-07

**Authors:** Ling Yi, Xin Jin, Jinghui Wang, Zhuohong Yan, Xu Cheng, Tao Wen, Bin Yang, Xiaojue Wang, Nanying Che, Zhidong Liu, Hongtao Zhang

**Affiliations:** ^1^ Department of Central Laboratory, Beijing Tuberculosis and Thoracic Tumor Research Institute, Beijing Chest Hospital, Capital Medical University, Beijing, China; ^2^ Department of Medical Oncology, Beijing Tuberculosis and Thoracic Tumor Research Institute, Beijing Chest Hospital, Capital Medical University, Beijing, China; ^3^ No. 2 Department of Thoracic Surgery, Beijing Tuberculosis and Thoracic Tumor Research Institute, Beijing Chest Hospital, Capital Medical University, Beijing, China; ^4^ Department of Pathology, Beijing Tuberculosis and Thoracic Tumor Research Institute, Beijing Chest Hospital, Capital Medical University, Beijing, China

**Keywords:** lung cancer, sCD137, CD137+ Tregs, negative regulation, CD137 agonist

## Abstract

Negative immune regulation plays a notable role in tumor immunity. This study aimed to confirm that CD137 mediates negative immunoregulation as well as agonist activity in tumor immunity. Soluble CD137 (sCD137), a prominent splice variant of membrane-bound CD137 (mCD137), was identified, and its concentration in the blood of lung cancer patients was increased. The baseline concentration of sCD137 in the blood was negatively correlated with the efficacy of neoadjuvant immunochemotherapy in a pilot study. The percentage of CD137+ regulatory T cells (Tregs) in the blood of lung cancer patients was also increased, and further enriched at the tumor site; Foxp3, CTLA-4, IL-10, IL-35-Ebi3, sCD137 and costimulatory molecules expression were also higher, indicating increased immunosuppressive activity. A high percentage of CD137+ Tregs in the tumor was associated with worse OS outcomes among patients with high CD137+CD8+ T cell infiltration levels. Notably, targeting CD137+ Tregs using an engineered CD137 agonist with wild-type mouse IgG2a Fc clearly decreased the total Treg numbers and eliminated the tumor in the CT26 model and prolonged the survival rate of a Lewis lung carcinoma (LLC) model. These results indicated it may be possible to empower CD137 agonist with ability to abolish CD137-mediated negative regulation to enhance its antitumor efficacy.

## Introduction

Checkpoint treatments, such as programmed cell death 1 (PD-1) and PD-1 ligand 1 (PD-L1) blocking monoclonal antibodies (mAbs), marked the beginning of the era of tumor immunotherapy. PD-1/PD-L1 antibodies primarily regulate CD8+ T cell function and exhibit broad-spectrum therapeutic efficacy (1). However, tumors demonstrate primary and acquired resistance, and the benefit of such antibodies is generally low, with only 12.46% of all cancers and approximately 20% of the major types of lung cancer responding to the antibodies (2–4). Unpredictable therapies may result in autoimmune or autoinflammatory reactions, termed immune-related adverse events (irAEs) (5). Ongoing clinical and basic research efforts aim to overcome these limitations and highlight the need for more effective and novel approaches. Recently, there has been noticeable progress in combining anti-PD-1/anti-CTLA-4 and anti-PD-L1/anti-TIGIT, which have an objective response rate of 30-50% in advanced non-small-cell lung cancer (NSCLC) (6, 7). Therefore, it is essential to identify new targets for immunomodulation to broaden the application of immunotherapy to more patients, including those with lung cancer and other tumors (8).

T cell proliferation and differentiation depend on the modulation of antigen receptor signaling by both costimulatory and coinhibitory receptors. CD40, OX40, ICSO and CD137 are the most likely costimulatory receptors involved in these processes because they are distributed in various immune-responsive cell subsets that coordinate the adaptive immune processes of T cell priming and expansion and memory T cell formation and survival (9). CD137 is an extremely attractive immunomodulatory target. Although CD137 is widely expressed, its expression in CD8+ T cells and natural killer (NK) cells is highly inducible; as these two cell types compensate for defects in the other type, activation of both leads to greater efficacy in the treatment of established tumors with low immunogenicity (10–12). The central roles of CD8+ T cells in contributing to CD137 costimulation, which promotes CD8+ T cell survival and expansion, and to the formation of memory T cells have been well defined in preclinical studies (13). However, the clinical trial results for CD137 antibodies are complicated (14). Although excessive immune stimulation was observed during cancer immunotherapy in an early clinical trial for the CD137 monoclonal antibody (mAb) urelumab, a low dose of urelumab is relatively safe (15, 16). Furthermore, when combined with rituximab, another antibody, utomilumab, exhibited acceptable systemic toxicity and good preliminary clinical activity in patients with CD20+ non-Hodgkin’s lymphoma, including rituximab-refractory patients (17). Recent studies have clarified that increased CD137 gene expression in tumors during immunotherapy with nivolumab and a CD137 agonist combined with PD-1 blockade results in robust antitumor immunity (18, 19). Trials studying the efficacy of anti-PD-1/PD-L1 combined with agonistic CD137 mAbs in solid tumors have been reactivated and are ongoing (20). Moreover, several newly designed antibodies against human CD137 are being developed (21, 22). Nevertheless, in order for CD137 antibodies to be successfully used in the clinic, the challenges related their toxicity and low antitumor immunomodulatory efficacy must be overcome.

Anti-CD137 therapeutic strategies are potentially limited by the expression of negative immunomodulatory factors other than the PD-1 and CTLA-4 on T cells. The present study clarified that CD137 itself mediates negative regulation and that the systemic levels of soluble CD137 (sCD137) and the percentage of CD137+ regulatory T cells (Tregs) are increased in lung cancer. A CD137 agonist while empowerded by targeting CD137-mediated negative regulation may show enhanced antitumor activity and may have utility in combination immunotherapy.

## Materials and Methods

### Patients and Specimens

Anticoagulant plasma was collected from 83 lung cancer patients (59 untreated and 24 treated with neoadjuvant immuno-chemotherapy) from 2018 to 2021 at Beijing Chest Hospital and 91 healthy donors (controls) and stored at -80°C for analysis of blood sCD137 levels. In addition, immune cell subsets in blood samples from 29 untreated patients, 34 healthy donors and 10 fresh tumor tissues were analyzed.

Another retrospective cohort containing 90 NSCLC tissues collected in 2013 at Beijing Chest Hospital and their representative tissue microarrays (TMAs) were included. The TMAs were prepared using two 1-mm diameter tumor cores from the formalin-fixed paraffin-embedded (FFPE) tissue block of each patient. All involved patients had received no treatment before surgery and had histologically confirmed lung cancer. The patients were followed up until January 2020 to evaluate overall survival (OS).

### Cloning of the Human sCD137 Gene and ARMS-QPCR

Patient peripheral blood mononuclear cells (PBMCs) were isolated and resuspended at a density of 1×10^6^/ml in 10% RPMI 1640 medium (Gibco) containing 50 μg/ml phytohemagglutinin (PHA, Sigma) for 24 h, 48 h or 72 h. Total RNA was isolated from stimulated cells using an RNA Easy Fast Tissue/Cell Kit (Tiangen Biotech). Then, CD137 was amplified using the primers with the following sequences: sense, 5’-gactgttgctttgggacattta-3’; antisense, 5’-tcacatcctccttcttcttcttc-3’. The obtained PCR products were cloned into the T-easy vector (Promega Corporation) for sequencing.

To detect sCD137 mRNA in different cell subsets, Tregs, non-Tregs, CD4+ T cells, CD8+ T cells, and CD4+CD25- T cells were isolated by using a FACSAia III Cell Sorter (BD Bioscience). The concentration of all cell subset samples was made equal for ARMS-QPCR, which was performed with GoTaq^®^ Probe qPCR Master Mix (Promega Corporation) and Cobas Z480 (Roche) according to the manufacturer’s instructions. The sequences of the primers and probes were as follows: sCD137: sense, 5’-atctgtcgaccctggacaaaga-3’; antisense, 5’-tcacatcctccttcttcttctt-3; probe, 5’6-FAM-ctggacaaagacactctccgcagatcat-TRMAR3’; β-actin: sense, 5’-cccagatcatgtttgagacctt -3’; antisense, 5’-gtggtggtgaagctgtagcc-3’; probe, catgtacgttgctatccaggctgtgct. Target gene expression was normalized to β-actin expression.

### Analysis of sCD137 Levels by ELISA

Costar ELISA plates (2 μg/ml) were coated with anti-human CD137 mAb (h41BB-M127, BD Pharmingen) and then blocked with 5% skim milk (BD Difco) for 2 h at 37 °C. Undiluted plasma from patients and healthy donors was incubated in the coated plates for 2 h at room temperature (RT). The captured sCD137 was detected using 2 μg/ml biotinylated-anti–CD137 mAb (4B4-1, BD Pharmingen) followed by incubation with streptavidin-HRP (1:5000, Cell Signaling Technology) for 0.5 h. The samples were developed using substrate reagents, and then stop solution (BD Biosciences) was added. The optical density (OD) was evaluated at 450 nm with a MULTISKAN GO instrument (Thermo Scientific). The plates were washed three times with 1× PBS containing 0.5% Tween-20 following every step, and all samples were assayed in duplicate. A standard curve was generated for each assay using recombinant human CD137-His (Sino Biological Inc).

### Analysis, Sorting, and Cultivation of T Cell Subsets

Blood Tregs and CD137+ Tregs were identified with a panel of antibodies, i.e., PerCP-anti-CD3 (SK7), APC-H7-anti-CD4 (PRA-T4), BV711-anti-CD25 (2A3), BV421-anti-CD127 (HIL-7R- M21), and PE-anti-CD137 (4B4-1). To analyze the percentage of Tregs and CD137+ Tregs in tumors, single-cell suspensions were prepared by cutting fresh tumor tissues into small pieces (2-4 mm) and then using a Tumor Dissociation Kit (human, Miltenyi Biotec GmbH) and GentleMACS device according to the manufacturer’s instructions. The obtained single-cell suspensions were washed, stained for 15 min with Fixable Viability Stain 440UV, and then incubated for 30 min with a set of antibodies, including PerCP-anti-CD3, PE-Cy7-anti-CD4 (SK3), BB515-anti-CD25 (2A3), APC-anti-CD127 (HIL-7R-M21), and BV421-anti-CD137 (4B4-1). Finally, erythrocytes (RBCs) were lysed for 15 min, and the stained cells were washed twice prior to analysis with an LSRFortessa flow cytometer (BD Biosciences). To isolate CD137+ Tregs and CD137- Tregs from tumor samples, single-cell suspensions were stained with Fixable Viability Stain 440UV for 15 min, washed twice and labeled with PerCP-anti-CD3, PE-Cy7-anti-CD4, BB515-anti-CD25, APC-anti-CD127, and BV421-anti-CD137 for 30 min as described above. After RBCs were lysed and the remaining cells were washed, the resuspended cells were sorted with a FACSAria III flow cytometer.

For analysis of Tregs and CD137+ Tregs in mice, blood cells were labeled with an anti-CD16/CD32 antibody (2.4G2, mouse Fc block), washed twice and labeled with PerCP-CY5.5-CD3 (145-2C11), FITC-CD4 (RM4-5), and BV421-CD137 (1AH2) for 30 min; then, RBCs were lysed for 15 min, and the remaining cells were fixed and labeled with APC-anti-mouse FOXP3 (clone FJK-16s, eBioscience). CD8+ T cells and CD137+CD8+ T cells were labeled with PerCP-CY5.5-CD3, FITC-CD8 (53-6.7), and BV421-CD137. Mouse tumor single-cell suspensions were prepared using a mouse tumor dissociation kit (Miltenyi Biotec GmbH) and Fixable Viability Stain 575V. Dissociated single-cell suspensions were labeled with anti-CD16/CD32 and the antibodies used for blood analyses. All antibodies used for flow cytometry analyses and lymphocyte sorting except anti-FOXP3 were purchased from BD Biosciences.

### Whole-Exome Sequencing and T Cell Receptor β Sequencing

For RNA-seq library preparation and sequencing, total RNA was extracted from CD137+ Tregs and CD137- Tregs using an RNeasy Micro Kit (QIAGEN GmbH). RNA quantity and purity were assessed using an Agilent 2100 bioanalyzer (Agilent Technologies). The total RNA was sent to the Beijing Genomics Institute for library construction following standard protocols. The library products were sequenced using a BGISEQ-500 instrument (BGI-Shenzhen, China). Standard bioinformatic analysis was performed by the Beijing Genomics Institute. Briefly, gene expression levels were quantified with RSEM (v1.2.12) after read cleaning and genome mapping. Differentially expressed genes (DEGs) were identified *via* DEseq2. Heatmaps were drawn using pheatmap (v1.0.8) according to gene expression levels in different samples. Genes with a Q value <0.05 were considered significantly differentially expressed. For immune repertoire library preparation and sequencing, 1 µg RNA samples were partitioned to construct a library for T cell receptor β (TRB) chain sequencing through a two-step PCR method. TRB genes were sequenced on a HiSeq 4000 instrument (Illumina, La Jolla, CA) using the standard paired-end 150 and paired-end 100 protocols. Base calling was performed according to the manufacturer’s instructions. The clone type frequency was analyzed using VDJ tools.

### Immunohistochemistry and Multiplexed Quantitative Immunofluorescence

Tumor infiltrating lymphocyte (TIL) subtypes were assessed *in situ* by multiplexed quantitative immunofluorescence (QIF) in TMA format using the PANO 6-Plex IHC Kit (Panovue, Beijing, China, 0003100100) according to the manufacturer’s instructions. Briefly, histological sections from the patients were deparaffinized and subjected to antigen retrieval; the sections were then stained with anti-CK antibodies to detect tumor epithelial cells; anti-CD4, anti-CD8, anti-Foxp3, and anti-CD137 antibodies to detect T lymphocytes; and 4′,6-diamidino-2-phenylindole (DAPI) to visualize the cell nuclei. The following primary antibodies were used: pan-CKs (ab27988, clone AE1/AE3, 1:500 dilution), CD4 (ab133616, clone EPR6855, 1:500), CD8 (ab17147, clone C8/144B, 1:200), Foxp3 (ab20034, clone 236A/E7, 1:200) and CD137 (ab252559, BLR051F, 1:500). The TMA slides were scanned with a Vectra multispectral microscope (Akoya Biosciences). Images of single-color-stained tissue sections were obtained, and the spectrum of each fluorophore was extracted to create the required spectrum for multiplex immunohistochemical staining, which was performed in InForm2.4.8. The densities of Tregs (FOXP3+ cells), CD137+ Tregs (CD137+FOXP3+ cells) and CD137+CD8+ T cells were calculated, and the data are presented as the percentage of positive cells among total cells in each selected TMA core. The stained TMA slides were visually examined by a pathologist, and areas with staining artifacts were excluded.

### Engineering and Preparation of Therapeutic Antibodies

A rat anti-mouse CD137 agonist (1D8, US patent #7,754,209 B2, SEQ ID No. 101 and 103) was engineered with chimeric mouse IgG2a (mIgG2a) (Wt-mAb). mIgG2a with FcγR mutations (Mut-mAb; D265A, N297A, L234A, L235A and P329A) was generated to eliminate *N*-linked glycosylation; these simultaneous mutations effectively reduce Fc binding to FcγR and antibody-dependent cell-mediated cytotoxicity (23). To obtain a potential local target in tumors using CD137, we prepared CD137×hEGFR (mouse CD137 and human EGFR)-bispecific antibodies with Wt-mAb and Mut-mAb formats using a eukaryotic expression system (GenScript Co. Ltd.; **Supplementary Figure 4A**). Two milligrams of each purified antibody was obtained using Protein A affinity chromatography.

### Therapeutic Studies in Mice

Female BALB/c and C57BL/6 mice were purchased from Beijing Vital River Laboratory Animal Technology Co. Ltd. All mice were maintained under specific pathogen-free conditions, housed at a controlled temperature and humidity and under a 12 h light/dark cycle. In the tumor treatment studies, 6- to 8-week-old female mice were subcutaneously implanted with 5×10^5^ CT26 cells (colon carcinoma cells, ATCC) or 1×10^6^ Lewis lung carcinoma cells (LLC cells,ATCC). Tumor area was monitored 6-7 days after tumor transplantation, and the mice were randomly divided into three treatment groups so that the mean tumors of the groups were similar. The mice were intratumorally injected with Wt-mAb or Mut-mAb (5 μg per mouse, diluted with 1×PBS) or PBS as a control 3 times at 2-day intervals. The tumor area was then measured every 3 days using electronic calipers. Blood and tumors were harvested on day 13 to analyze T cell subsets before and after treatment. The mice were sacrificed when the tumor area reached 225 mm^2^. The percentage of mice that survived to the end point was calculated. Each experiment was repeated three times.

### Statistical Analysis

Data analyses were performed using GraphPad Prism 6.0. The Mann–Whitney test was used to compare non-normally distributed data between two groups, and unpaired t test with Welch’s correction was used to compare normally distributed data with unequal variances between two groups. Unpaired t test was used to compare normally distributed data with equal variances. One-way ANOVA followed by Tukey’s post-hoc multiple comparisons test was used to compare normally distributed data with equal variances between multiple groups. The Kruskal–Wallis test followed by Dunn’s post-hoc test was used to compare normally distributed data with unequal variances. OS at the end point was compared using Kaplan–Meier estimates and the log-rank test, and P < 0.05 was considered statistically significant.

## Results

### High Blood sCD137 Levels in Patients With Lung Cancer

**Figure 1:**
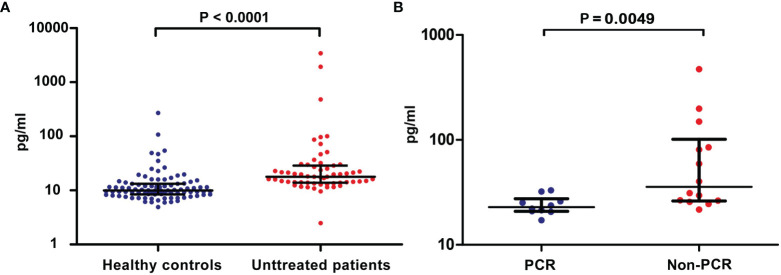
Levels of blood sCD137 in patients with lung cancer. **(A)** sCD137 levels in blood samples from 91 healthy controls and 59 untreated patients. **(B)** Baseline blood sCD137 levels in neoadjuvant immunochemotherapy-treated patients with a pathological complete response (PCR) and neoadjuvant immunochemotherapy-treated patients without pathological complete response (Non-PCR). Differences are indicated as P values. The error bars represent the IQRs.

Considering that sCD137 negatively regulates CD137 signaling, we initially investigated the level of free sCD137 in the peripheral blood of 59 untreated lung cancer patients and 91 healthy donors. The sCD137 concentration ranged from 2.47 to 3431.59 pg/ml (mean=121.85 pg/ml, median=17.80 pg/ml) and 4.91 to 270.58 pg/ml (mean=16.37 pg/ml, median=9.974 pg/ml) in cancer patients and healthy donors, respectively. sCD137 levels in the peripheral blood of lung cancer patients were 7.4 times higher than those in the peripheral blood of healthy controls (**Figure 1A**, P<0.0001, median ± IQR). The sCD137 level was not correlated with age, pathological type or clinical stage (**Table 1**). Then, sCD137 levels in 24 lung cancer patients who received neoadjuvant immunochemotherapy were tested, and it was found that there was a significant difference in baseline sCD137 levels between patients with a pathological complete response and patients without a pathological complete response patients. Patients with low baseline sCD137 levels were more inclined to achieve a pathological complete response (**Figure 1B**, P=0.0049, median ± IQR). Twenty-four patients had 18 squamous cell carcinoma, 5 had adenocarcinoma, and 1 had sarcomatoid carcinoma; 20 were stage III, and 4 were stage IB~IIB.

**Table 1:** Correlations between blood sCD137 levels and clinical characteristics in untreated patients.

Variables	Total	sCD137	P value
		(pg/ml)	
Healthy controls	91	16.37	<0.0001
Lung cancer patients	59	121.85	
Lung cancer patients			
Age			0.0585
<60	21	42.17	
≥60	38	165.88	
Stage			
I+II	22	203.58	0.5887
III+IV	37	73.24	
Type			0.1914
Squamous cell carcinoma	17	165.23	
Adenocarcinoma	34	122.80	
Small cell carcinoma	8	25.60	

### sCD137 Gene Cloning and Constitutive Expression in Tregs

**Figure 2:**
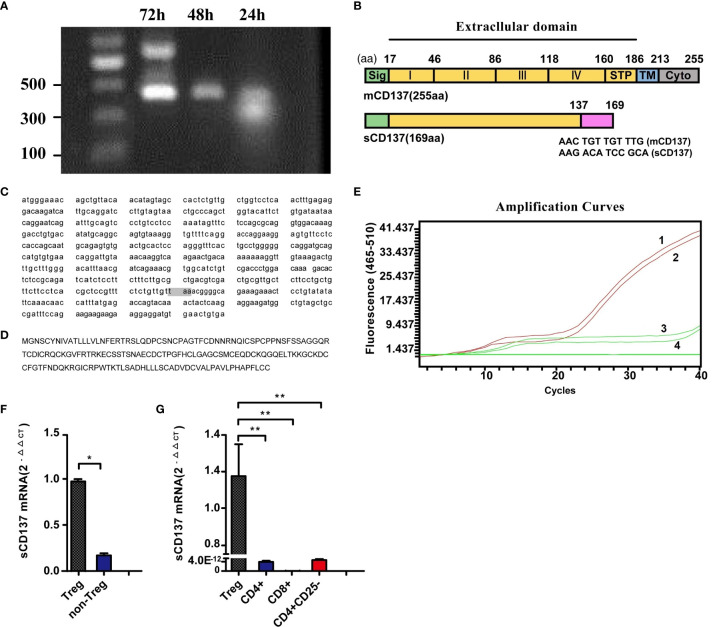
Cloning of sCD137 and constitutive sCD137 expression in Tregs. **(A)** Amplification of mCD137 and sCD137 from activated PBMCs by RT–PCR. **(B)** Alignment of sCD137 to full-length mCD137. The deletion in sCD137 is indicated at the amino acid level, and the codon changes caused by the deletion are shown at the splice point. STP, region rich in the amino acids serine, threonine and proline. **(C)** The whole sequence of sCD137. The shaded “taa” codon is the stop codon. **(D)** The predicted amino acid sequence of sCD137. **(E)** Identification of specific primers and probes for amplifying and detecting sCD137 by ARMS-QPCR. Only sCD137 was amplified. Amplification curves 1 and 2 are for T-easy-sCD137, and amplification curves 3 and 4 are for T-easy-mCD137. **(F)** sCD137 mRNA comparison of non-Tregs and Tregs. **(G)**, sCD137 expression in unstimulated Tregs, CD4+ T cells, CD8+ T cells and CD4+CD25- T cells. *P< 0.05, **P< 0.01.

To explore the source of sCD137 in resting lymphocytes, sCD137 mRNA was cloned by RT–PCR. The primers are shown in **Supplementary Figure 1** (the sequences are unlined with a wavy line). We obtained two products after PCR amplification (**Figure 2A**, 24 h lane). Nucleotide sequencing revealed a larger product with a molecular weight of 408 bp and a smaller product with a molecular weight of 276 bp. The shorter region contained a 132-base deletion (414 to 545, indicated by the black brackets, **Supplementary Figure 1**), which was predicted to encode the cysteine-rich domain IV (CRDIV) and STP region of membrane-bound CD137 (mCD137) (**Figure 2B**). This deletion resulted in a frame shift, generating a “taa” translational stop codon 96 nucleotides downstream (**Supplementary Figure 1**). This CD137 isoform consisted of 169 amino acids and excluded the transmembrane region found in sCD137 (**Figure 2B**). When the RT–PCR products were inserted into the T-easy vector for sequencing, 8 of 9 colonies were identified as mCD137, and 1 colony was identified as sCD137. The full-length sequence of sCD137 and the predicted amino acid sequence are shown in **Figures 2C, D**.

sCD137 expression was then analyzed using ARMS-QPCR. The primers and probe were confirmed to specifically amplify sCD137 (**Figure 2E**). The expression of sCD137 mRNA in Treg (△CT =17.02) and non-Treg (△CT =19.46) subsets was directly compared (P=0.0082; **Figure 2F**). The data showed that in resting T cell subsets, Tregs expressed sCD137 (**Figure 2G**), but CD4+ T, CD8+ T, and CD4+CD25+ T cells showed no sCD137 expression.

### Expanded Blood CD137+ Tregs in Lung Cancer Patients

**Figure 3:**
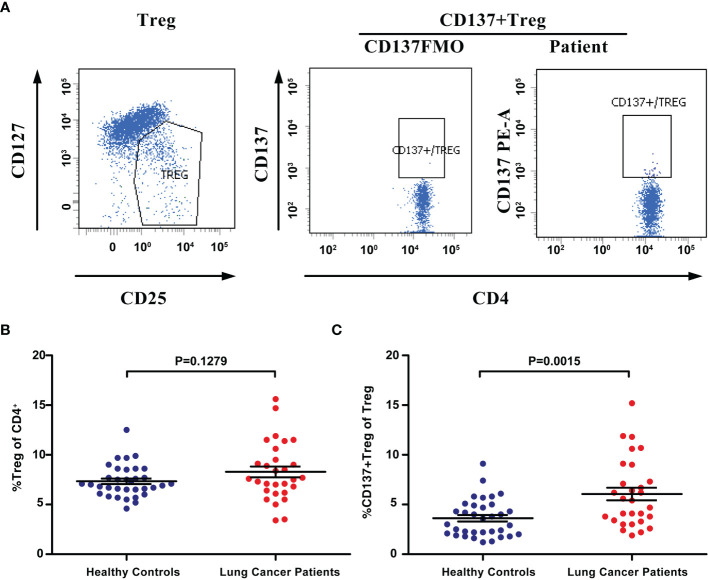
The percentage of CD137+ Tregs was increased in the blood of lung cancer patients. **(A)** Tregs and CD137+ Tregs were gated by flow cytometry. **(B)** The percentage of Tregs in lung cancer patients (n=29) and healthy controls (n=34). **(C)** The percentage of CD137+ Tregs in the same patients and healthy controls. Differences are indicated as P values. The error bars represent the SEMs.

Few blood-derived CD4+ T cells expressed CD137 in healthy controls and lung cancer patients (**Supplementary Figure 2**). CD137 expression in the Treg population in the peripheral blood was analyzed (**Figure 3A**). FACS analysis revealed that the percentage of Tregs (CD4+CD25+CD127^low^) among all CD4+ T cells ranged from 3.4% to 15.6% (mean, 8.29%) in 29 lung cancer patients and 5.2% to 12.5% (mean, 7.34%) in 34 healthy controls. The percentage of peripheral blood Tregs was not significantly higher in patients than in healthy controls (P=0.1279, **Figure 3B**). However, the percentage of CD137+ Tregs ranged from 1.9% to 15.2% in patients (mean, 6.06%) and from 1.2% to 9.1% (mean, 3.62%) in healthy donors; thus, the percentage of CD137+ Tregs was significantly increased in lung cancer patients compared to healthy controls (P=0.0015, **Figure 3C**). No correlation was found between the percentage of Tregs or CD137+ Tregs and the clinical stage or pathological type (**Supplementary Table 1**).

### CD137+ Tregs Were Further Enriched in Tumors With a Prominent Treg Phenotype

**Figure 4:**
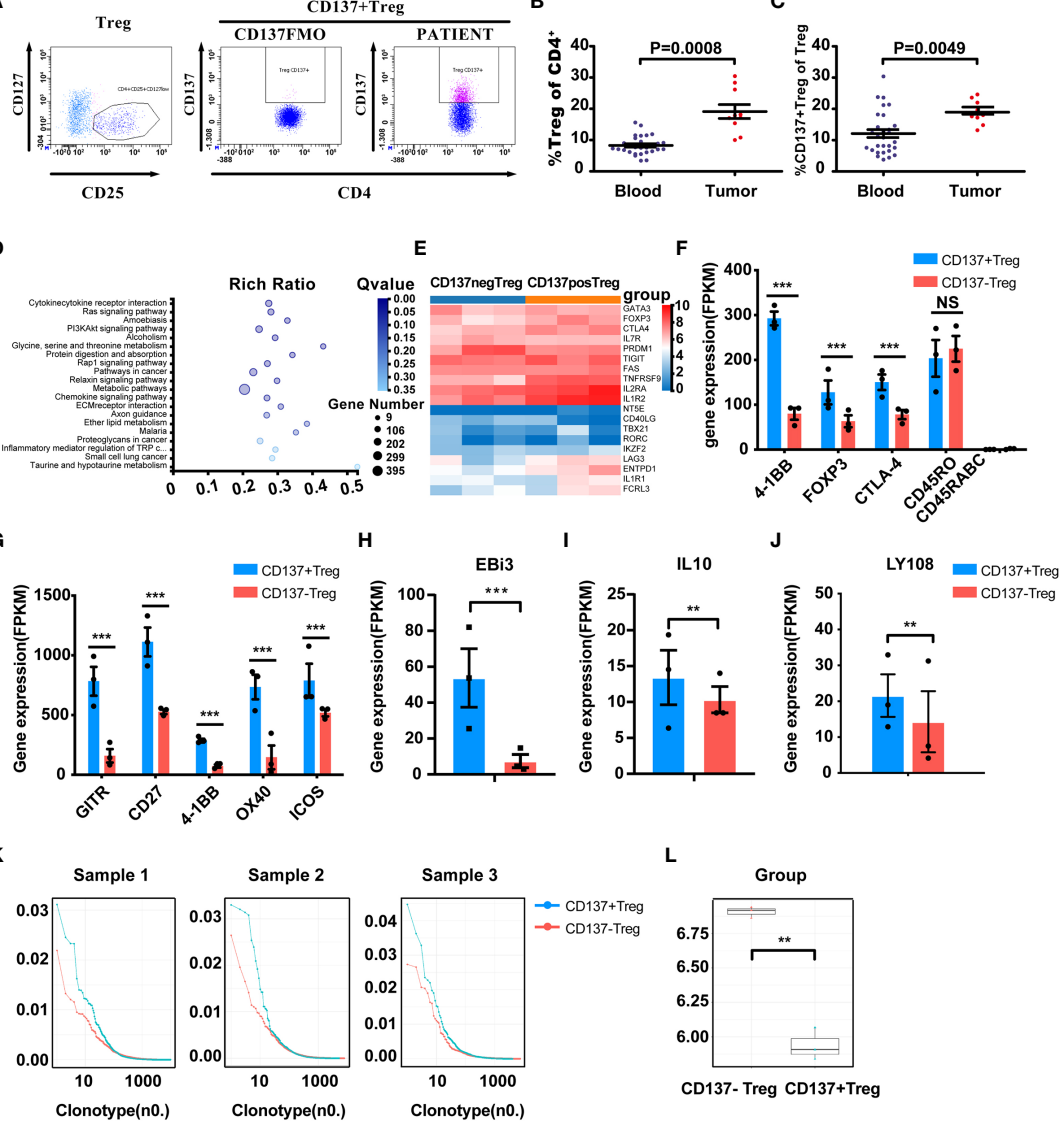
Enrichment and characterization of CD137+ Tregs in the tumor microenvironment. **(A)** Gated CD137+ Tregs derived from human cancer tissues *via* FACS; middle panel, CD137+ Tregs FMO control. **(B, C)** Treg and CD137+ Treg infiltration in tumors (n=10, unpaired samples) compared with the blood of lung cancer patients. P values are shown. **(D)** PI3K-AKT pathway-related molecules were differentially expressed in the CD137+ and CD137- Treg populations according to KEGG pathway analysis. **(E)** Comparative heatmap of the expression of Treg-associated markers, including transcription factors, in CD137+ Tregs (orange) and CD137- Tregs (blue). **(F)** The expression of eTreg-related markers in CD137+ Tregs and CD137- Tregs. **(G)** The expression of activated T cell markers in CD137+ Tregs and CD137- Tregs. **(H–J)** Differential expression of EBi3, IL-10 and LY108 in CD137+ Tregs and CD137- Tregs. ***Q value < 0.001, **Q value < 0.01. **(K)** The TCR clone frequency in CD137+ Tregs and CD137- Tregs in three individual samples was compared after TCB sequencing. **(L)**, TCR clone diversity in the two groups, **P< 0.01. The error bars represent the SEMs.

The proportions of Tregs and CD137+ Tregs in fresh tumor tissues from 10 patients were analyzed by FACS (**Figure 4A**). The characteristics of these 10 patients and the Treg and CD137+ Treg cell proportions in tumors are shown in **Table 2**. The percentages of Tregs and CD137+ Tregs ranged from 10.8% to 30.45% (mean, 19.13%) and from 6.6% to 12.3% (mean, 9.47%), respectively. Interestingly, the percentages of Tregs and CD137+ Tregs were significantly increased in tumor samples compared to blood samples (Tregs: 19.13% vs. 8.286%, P=0.0008; CD137+ Tregs: 9.47% vs. 6.05%; P=0.0049; **Figures 4B, C**).

**Table 2:** Tumor sample characteristics and CD137+ Treg infiltration.

	Sex	Age	Tumor size	Tumor type	Stage	% of Tregs among CD4+ T cells	% of CD137+ Tregs among Tregs
1	M	48	1.8*1.2*1.0	A	T1bN0M0	10.8	11.8
2	F	46	2.5*1*1	A	T1N0M0	18.15	9.8
3	F	44	3.3*2*1.8	A+S (<5%)	T2aN0M0	10	8.6
4	F	61	1.6*1.5*1.2	A	T1bN2M0	17.85	12.3
5	M	67	2.5*1.5*1	A	T2N0M0	28.35	8.8
6	F	66	2.3*1.8*1.8	A	T1aN0M0	17	7.4
7	F	69	6*4*3.8	AS	PT3N0M1b	30.45	9.4
8	F	76	2.1*2*1.8	A	T1CN0M0	17.1	11
9	F	75	3.1*3*1.5	A	T2N0M0	15.4	9
10	M	65	4.2*3*4	C	T2N0M0	26.15	6.6

M, male; F, female; A, adenocarcinoma; C, carcinosarcoma; A+S, adenocarcinoma mixed with small cell lung cancer; AS, adenosquamous carcinoma.

CD137+ Tregs and CD137- Tregs from 3 individual tumor tissues were sorted by FACS. Treg-related gene expression was analyzed by transcriptome sequencing. The heatmap of the PI3K-Akt signaling pathway shows highly ranked differences between CD137+ Tregs and CD137- Tregs (Q value=0.0038; **Figure 4D**); furthermore, Akt expression was 2.59 times higher in CD137- Tregs than in CD137+ Tregs. The expression of essential Treg-associated markers, including transcription factors such as Foxp3, CD25, CTLA-4, TIGIT and LAG3, was further upregulated in CD137+ Tregs (**Figure 4E**). Coexpression of CD25, CTLA-4, and Foxp3, which are related to effector Tregs (eTregs), was higher in CD137+ Tregs than in CD137- Tregs (**Figure 4F**), and the expression of GITR, CD27, CD137 and OX40 was upregulated to a greater extent in CD137+ Tregs (**Figure 4G**). Furthermore, CD137+ Tregs expressed higher IL-10 and Ebi3 levels than CD137- Tregs (1.3 times and 7 times higher, respectively, **Figures 4H, I**). T cell receptor (TCR) sequencing analysis of three samples revealed single-clone expansion in the CD137+ Treg population (**Figures 4K, L**). In these three samples, LY108 expression was significantly higher in CD137+ Tregs than in CD137- Tregs (1.5 times, **Figure 4J**).

### A High Percentage of CD137+ Tregs in Tumors Was Associated With Worse OS

**Figure 5:**
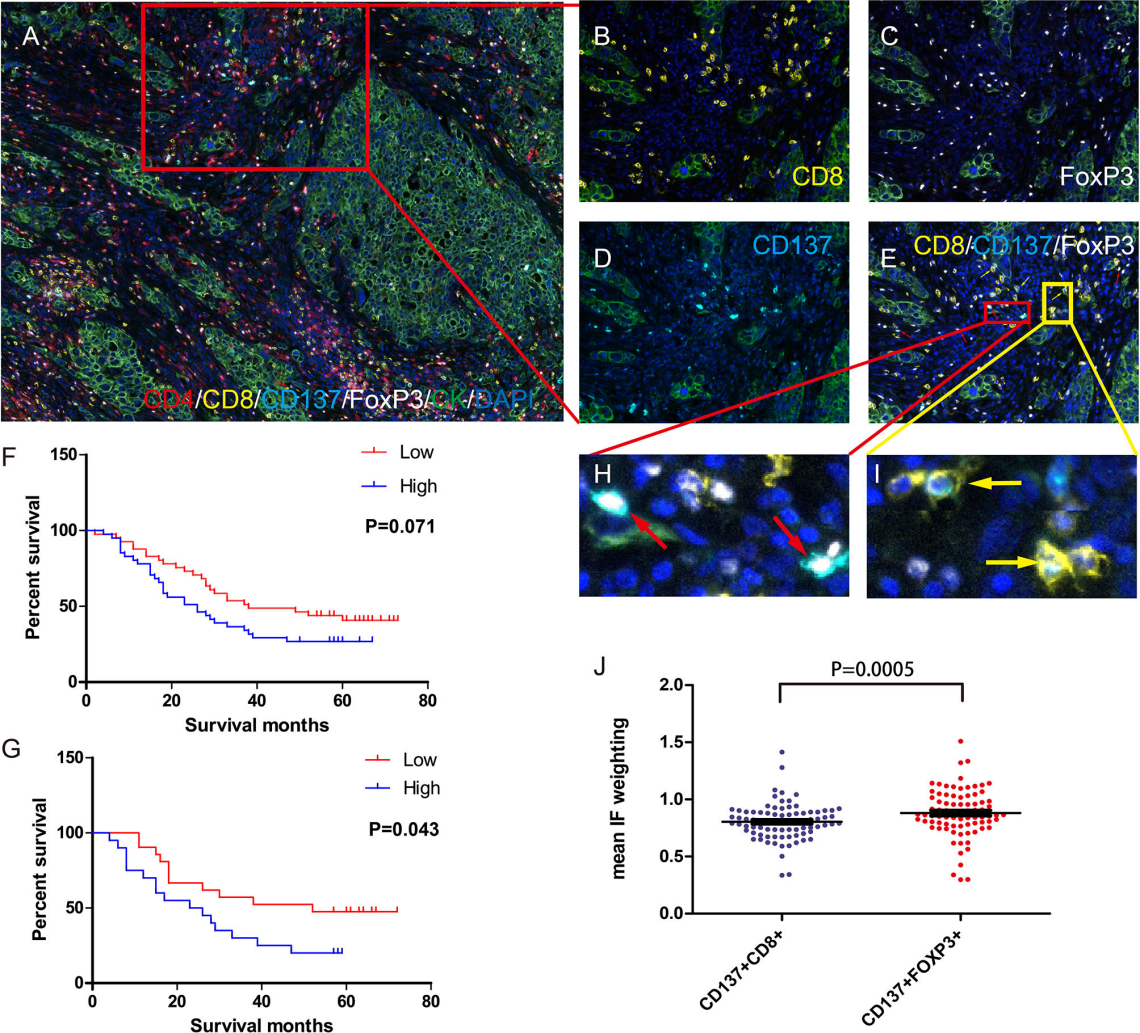
Density of Foxp3+ and CD137+FOXP3+ cells in the tumor microenvironment and its correlation with prognosis. **(A)** Immune cells in the tumor microenvironment of a TMA core (20×). Magnified view of the red box: CD8+ T cells **(B)** Foxp3+ cells **(C)** CD137+ cells **(D)** and double-positive cells. **(E)** CD137+CD8+ cells are indicated by the yellow arrows, and CD137+Foxp3+ cells are indicated by the red arrows. **(F)** Impact of CD137+Foxp3+ cell density on patient OS. Dichotomization was based on the median; patients in the low-density group are indicated by the red line, and patients in the high-density group are indicated by the blue line. Log-rank *P* values are shown for each graph. **(G)** Correlation between CD137+Foxp3+ cell density and OS in patients with a high number of infiltrating CD137+CD8+ cells in the tumor microenvironment. CD137+CD8+ cell dichotomization was also based on the median. **(H, I)** IF intensity of CD137+FoxP3+ cells and CD137+CD8+ cells. **(J)** The mean IF intensity weighting of CD137 in CD137+CD8+ cells and CD137+FoxP3+ cells in TMA cores from 82 lung cancer patients. Differences are indicated as P values (paired t test). The error bars represent the SEMs.

TMA samples from 82 qualified patients were subjected to QIF with a Vectra multispectral microscope. Of the 82 patients, 35 had adenocarcinoma, 47 had squamous cell carcinoma, 49 were in stage I+II, 33 were in stage III+IV, 65 had died by Jan 2020, and 17 were alive in Jan 2020. The patients’ clinical characteristics are shown in **Supplementary Table 2**. The density and spatial distribution of the CD4+, CD8+, Foxp3+, CD137+Foxp3+, and CD137+CD8+ T cell subsets are shown in **Figures 5A–E**.

We calculated OS from the date of surgery to death. Analysis of the association between OS and CD137+ Treg (CD137+FOXP3+) cell density in the tumor microenvironment revealed that patients with a higher CD137+ Treg density had a worse OS (P=0.0714, **Figure 5F**). Regarding tumor microenvironments with a high density of activated CD137+CD8+ T cells, patients with a high CD137+ Treg density had a worse OS than those with a low CD137+ Treg density (P=0.043, **Figure 5G**). Moreover, the mean IF intensity of CD137 was significantly higher in CD137+ Tregs than in CD8+ T cells (P=0.0005, **Figures 5H–J**). Similarly, patients with a high Treg (FOXP3+) density in the tumor microenvironment had a worse OS (**Supplementary Figure 3**).

### Modulation of Antitumor Efficacy by Targeting CD137+ Tregs With a CD137-IgG2a Agonist

**Figure 6:**
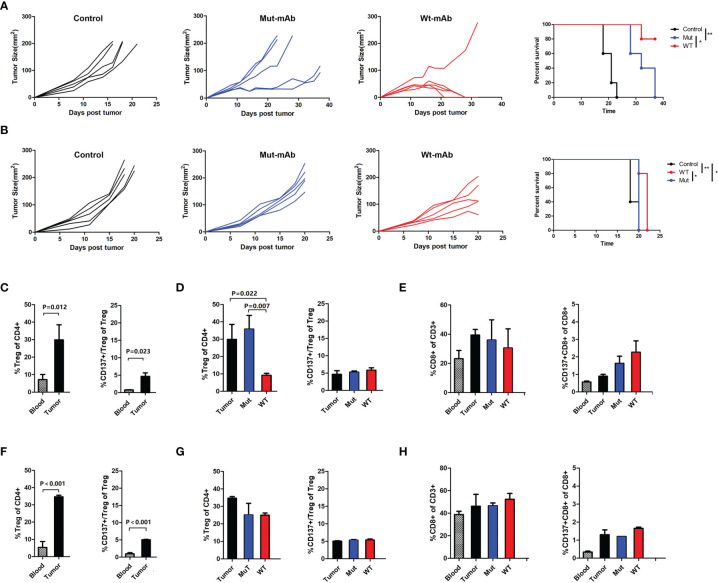
Targeting CD137+ Tregs with Wt-mAb enhanced antitumor efficacy *in vivo*. **(A)** CT26 (colon carcinoma) cells and **(B)** LLC cells treated with PBS (control) or the anti-CD137 mAbs Mut-mAb (with Fc mutation) and Wt-mAb (without Fc mutation). Mice (5 per group) were subcutaneously transplanted with CT26 or LLC cells. Six to 7 days later, when the tumor reached 0.5-0.7 cm in the largest diameter, the mice were injected intratumorally with mAbs (5 μg per mouse) 3 times at 2-day intervals. Tumor growth and mouse survival were monitored after transplantation. **(C)** The percentages of Tregs and CD137+ Tregs in blood and tumors from mice in the CT26 cell transplantation group and the changes in these percentages before and after mAb treatment **(D)**. **(E)** CD8+ T and CD137+ CD8+ T cell percentages in blood and tumors were compared before and after treatment. **(F–H)** The changes in the frequencies of T cell subsets were analyzed in mice in the LLC cell transplantation group as described for the CT26 cell transplantation group in **(C–E)**. A representative result of repeated experiments. The error bars represent the SEMs. *P < 0.05, **P < 0.01.

To analyze whether CD137+ Treg depletion can enhance antitumor immunity in solid tumors, chimeric rat anti-CD137×hEGFR (mouse CD137 and human EGFR)-bispecific wild-type IgG2a antibody (Wt-mAb) and Fc-silenced mutant IgG2a antibody (Mut-mAb) were generated (**Supplementary Figure 4A**). We verified the anti-CD137 Fc activity of the bispecific mAbs. The two mAbs retained equivalent specific binding to mouse CD137 (**Supplementary Figure 4B**). The therapeutic potential of these CD137 mAbs was evaluated in two mouse tumor models, an LLC cell transplantation model and a CT26 cell transplantation model. The Wt-mAb exerted a considerable therapeutic effect in the CT26 cell transplantation model, as 80% of the mice were tumor free at the long-term survival endpoint; in contrast, the Mut-mAb delayed the growth of CT26 cells but did not eradicate the tumors (**Figure 6A**). Both mAbs failed to confer a significant long-term benefit in the LLC cell transplantation model, but the tumors grew more slowly in the Wt-mAb-treated group (**Figure 6B**). The percentages of Tregs and CD137+ Tregs were significantly higher in the tumor microenvironment than in peripheral blood (**Figures 6C, F**). Importantly, the total number of Tregs was decreased in both models (**Figures 6D, G**, left) and was significantly decreased in the colon carcinoma model after Wt-mAb treatment (*P=*0.007, **Figure 6D**, left); however, the CD137+ Treg/Treg ratio was unchanged (**Figures 6D, G**, right). Treatment did not markedly affect the percentage of CD8+ T cells (**Figures 6E, H**).

## Discussion

The present study focused on the negative regulation of tumor immunity mediated by CD137. We initially observed markedly elevated blood sCD137 levels in lung cancer patients. An increased in the blood sCD137 level, which may reflect activation of immune cells, is frequently seen in immune-related diseases (24, 25), and such an increase can be observed in patients in which tumor immunotherapy is effective (17). We confirmed that sCD137 was primarily derived from activated T cells and that a high level of sCD137 correlated with the induction of CD137 expression on the cell membrane in vitro (**Supplementary Figure 5**). Our study also found that among unstimulated T lymphocytes, only Tregs expressed sCD137 (**Figure 2G**). Consistently, Ridgway WM showed that Tregs constitutively express CD137, whereas effector T cells express CD137 only briefly after activation, and that CD137+ Tregs are the primary cellular source of sCD137 (26, 27). Although the source of blood sCD137 in tumor patients needs to be further investigated, sCD137 is potentially derived from CD137+ Tregs in the tumor microenvironment, where most antitumor immune cells are in a suppressed state. sCD137 may be generated in two ways: alternative splicing of mRNA or cleavage of mCD137. We further confirmed that the source of sCD137 was alternative splicing of mRNA. Functionally, sCD137 binds to CD137 ligand (CD137L) and abrogates ligand-mediated activity in a manner dependent on its own ligand-binding domain (28, 29). In addition, hypoxia-induced secretion of sCD137 from tumor cells blocks CD137-CD137L costimulation to achieve immune escape (30). A recent study showed that a high level of sCD137 predicts a poor response to immunotherapy (31), which is consistent with our finding that patients with low baseline sCD137 levels were more likely to respond to neoadjuvant immunochemotherapy.

Tregs play a central role in maintaining immune tolerance and immunopathogenesis and functionally suppress various types of effector lymphocytes, such as CD4+ T helper cells and CD8+ cytotoxic T cells (32, 33). Tregs are one of the main escape mechanisms of tumor immunity (34). Compared with low FOXP3 mRNA expression, high FOXP3 mRNA expression is associated with a significantly worse prognosis in many types of tumors, and Foxp3+ T cell infiltration predicts a poor outcome in NSCLC (35–37). In our cohort of 82 patients, the presence of Foxp3+ T cells in the tumor microenvironment had the same predictive ability for OS. Furthermore, the correlation between the percentage of CD137+ Tregs and OS was dependent on the CD137+CD8+ T cell density, suggesting that CD137+ Tregs have an inhibitory effect on CD137+CD8+ T cells. Recently, Freeman ZT et al. reported a correlation between CD137/Foxp3 expression and prognosis in a variety of tumors (38). We found that the percentage of CD137+ Tregs was significantly increased in the blood of lung cancer patients and further enriched at tumor sites. A series of studies indicated that CD137+ Tregs have greater inhibitory activity in allergic immune regulation (35). We speculate that CD137+ Tregs may represent a major functional Treg population involved in tumor immunity. There are several mechanisms of Treg cell-mediated suppression, including IL-2 deprivation, secretion of inhibitory cytokines such as IL-10 and IL-35 (36), and acquisition of costimulatory molecules from antigen-presenting cells (APCs) via high-affinity binding to CTLA-4 (30). Consistent with these mechanisms, we determined that IL-10, IL-35-Ebi3, CD25 (IL-2Ra) and CTLA-4 mRNA levels were 1.3, 7, 2.13, and 1.94 times higher, respectively, in CD137+ Tregs than in CD137- Tregs, and that intratumoral Tregs exhibited an eTreg phenotype (CD45RA-Foxp3^hi^CD25^hi^CD4+), which has been demonstrated to be effector-like and highly suppressive (39–41). In addition, PI3K-Akt signaling was enhanced in CD137- Treg cells; this pathway has been shown to be closely related to Treg function (42). We also confirmed the occurrence of TCR clustering in the CD137+ Treg subset; in three tumor samples, this clustering was much higher in this subset than in the CD137- Treg subset. Antigen-specific Tregs have stronger inhibitory activity, especially in models of allergy and transplant rejection (43, 44). Single-cell sequencing revealed a striking bimodal distribution of CD137+ Tregs among CTLA4+ Tregs in NSCLC, which suggests that CD137+ Tregs are antigen-experienced and the main functional Tregs in tumors (45).

Antitumor immune responses can potentially be unleashed by inhibiting or depleting immunosuppressive factors, and methods that target the PD-1 pathway have achieved extensive clinical responses. Tregs, myeloid-derived suppressor cells (MDSCs) and tumor-associated macrophages (TAMs) exhibit relatively well-defined immunosuppressive functions at the cellular level (46, 47). Tregs are generally regarded as one of the major obstacles to successful clinical immunotherapy. We investigated the possibility of increasing the antitumor efficacy of targeting CD137+ Tregs via intratumoral injection, which was initially demonstrated to be a powerful method with limited side effects (11, 12, 48). Eliminating Tregs with CD137 mAbs increased the antitumor effect in two tumor models, especially the CT26 cell transplantation model. It has been reported that the mechanism primarily involves the activation of macrophages by IgG-Fc, which mediates antibody-mediated cell-dependent phagocytosis (ADCP) of Tregs (49–51). Regarding the lack of change in the percentage of CD137+ Tregs after Wt-mAb treatment, there is evidence that tumor-resident Tregs exhibit tumor neoantigen reactivity, which leads to their activation and clonal expansion in the tumor microenvironment, perhaps indicating that CD137 signaling may be essential for controlling the population of Tregs and their transient expansion. Consistent with this evidence, our data showed that the expression of LY108 on CD137+ Tregs was increased, indicating that these cells exhibited stronger proliferation ability and subsequent expansion (52). Therefore, targeting CD137+ Tregs effectively limited the total numbers of both Tregs and CD137+ Tregs and may have also reduced sCD137 secretion by CD137+ Tregs in tumors. We further confirmed that CD137 expression was higher in CD137+ Treg cells than in CD137+CD8+ T cells in the microenvironment, which clearly indicates the preferential binding of the CD137 mAb to CD137+ Treg cells, leading to functional Treg deletion by mAb-Fc, in lung cancer patients. In addition, previous work has shown that FOXP3 binds the 4-1BB promoter and that CD137 expression is upregulated in stimulated Tregs (49, 53).

However, it is important to maintain sufficient CD8+ T cells when conducing CD137 mAb-mediated ADCP (49). It has been widely demonstrated in preclinical strongly immunogenic, poorly immunogenic and spontaneous tumor models that CD137 antibodies mainly exert antitumor effects by activating CD8+ T cells (10–12). In this study, CD137 agonist when given IgG2a-Fc function targeting CD137+Tregs, clearly decreased total Treg numbers and maintained CD8+T cell numbers,which effectively enhanced the anti-tumor immune response. An expanded study exploring the effects of CD8+ T cells is necessary. There are other limitations in this study: Tregs inTMAs were only identified by Foxp3 markers, but not simultaneously analysed by CD4 and CD25; in preclinical tumor models, one experiment with 5 mice per group was not robust enough, more mice should be included, paticularly a validation in expanded mouse tumor models is necessary.

We conclude that strategies aimed at delivering CD137 antibodies to local tumors, such as the use of bispecific antibodies (53), may avoid unexpected systemic toxicity. In the development of next-generation antibodies, it may be possible to empower CD137 antibodies with Treg-depleting properties to potentially enhance the efficacy of immunomodulatory therapy for lung cancer and other types of cancer. Our bispecific mAb may be used to target human EGFR-positive tumors and locally regulate CD137 signaling in tumor immunotherapy studies.

## Data Availability Statement

The raw data are available in the National Genomics Data Center (https://bigd.big.ac.cn/gsa-human/) under accession number HRA000890.

## Ethics Statement

The present study involved the collection of blood from healthy humans, patients and mice and tumor tissue from patients and mice, and all related protocols were approved by the Beijing Tuberculosis and Thoracic Tumor Research Institute Ethics Committee. The animal experiments were conducted following relevant national and international guidelines.

## Author Contributions

Conception and design: HZ, ZL. Acquisition of data: LY, XJ, JW, ZY, XC, TW, XW, BY, NC. Analysis and interpretation of the data: HZ, LY. Writing, review, and/or revision of the manuscript: HZ, LY. Study supervision: HZ. All authors contributed to the article and approved the submitted version.

## Funding

This work was supported by the National Natural Science Foundation of China (grant numbers 81273209 and 30572123), Research on Key Technologies of Lung Cancer Diagnosis and Biotherapy in Beijing Health Planning Commission (PXM2018_026271_000002), and Beijing Municipal Administration of Hospitals Incubating Program (Code: PX2021062).

## Conflict of Interest

The authors declare that the research was conducted in the absence of any commercial or financial relationships that could be construed as a potential conflict of interest.

## Publisher’s Note

All claims expressed in this article are solely those of the authors and do not necessarily represent those of their affiliated organizations, or those of the publisher, the editors and the reviewers. Any product that may be evaluated in this article, or claim that may be made by its manufacturer, is not guaranteed or endorsed by the publisher.

## Supplementary Material

Supplementary Information Title: CD137 agonists targeting CD137-mediated negative regulation show enhanced antitumor efficacy in lung cancer.

Supplementary Figure 1 mCD137 cDNA sequence and primer sequences. The entire sequence of mCD137 cDNA. The straight underline indicates the transmembrane region. RT–PCR primers (wavy underline) used to amplify sCD137 from activated PBMCs yielded one splice variant that lacked nucleotides 414 to 545 (indicated by brackets). The frame shift results in a stop codon, i.e., the shaded “taa” codon. The sequences of the primers used for ARMS-QPCR are indicated in bold font, and the sequence of the probe is shaded.

Supplementary Figure 2 Percentage of CD137+CD4+ T cells in the blood. **(A)** Gated CD3+, **(B)** CD4+, and **(C)** CD137+CD4+ T cells. **(D)** The percentage of CD137+CD4+ T cells in healthy controls and lung cancer patients. The error bars represent the SEMs.

Supplementary Figure 3 Correlation of Treg+ cell density in the tumor microenvironment with OS. Foxp3+ cells in the tumor microenvironment in TMAs from 82 lung cancer patients were detected by multiplexed QIF. **(A)**, Impact of Foxp3+ cell density on patient OS. Dichotomization was based on the median: red line, patients in the low-density group; blue line, patients in the high-density group. Log-rank P values are shown for each graph. **(B)**, Correlation of Foxp3+ cell density with OS in patients with a high number of infiltrating CD137+CD8+ cells in the tumor microenvironment. CD137+CD8+ cell dichotomization was based on the median.

Supplementary Figure 4 Structure and specificity of the therapeutic antibodies. **(A)**, Structural diagram of the therapeutic antibodies used in this study. A wild-type CD137 mAb (Wt-mAb, mIgG2a) and mutant CD137 mAb (Mut-mAb, mIgG2a) with D265A, N297A, L234A, L235A and P329A mutations in FcγR were created via gene synthesis and expressed using a eukaryotic expression system. **(B)**, Binding of therapeutic antibodies to mouse CD137-His. The error bars represent the SEMs.

Supplementary Figure 5 Association of mCD137 protein expression with sCD137 protein expression. **(A)**, mCD137 expression and sCD137 levels in cell-free culture supernatant from non-Tregs and Tregs cultured under the indicated cell culture conditions for 3 days. **(B)**, mCD137 expression on CD4+ T subsets grown under the indicated culture conditions and sCD137 levels in the corresponding cell-free culture supernatant. **(C)**, mCD137 expression on CD8+ T subsets grown under the indicated culture conditions and sCD137 levels in the corresponding cell-free culture supernatant. Unstimulated, complete medium only; IL-2, complete medium + IL-2 (100 U/ml); CD28 mAb, complete medium + anti-human CD28 mAb (1 μg/ml); CD3 mAb, complete medium + anti-human CD3 mAb (OKT3, 1 μg/ml); IL-2+CD28+CD3, complete medium + IL-2 + anti-CD28 + anti-CD3; Treg, Treg + complete medium + IL-2 + anti-CD28 + anti-CD3. One representative assay of three experiments is shown.

## Abbreviations

mAbs, monoclonal antibodies; mCD137, membrane-bound CD137; sCD137: soluble CD137; Tregs, regulatory T cells; PD-1, programmed cell death 1; PD-L1, PD-1 ligand 1; NSCLC, non-small cell lung cancer; irAEs, immune-related adverse events; TCRs, T cell receptors; PBMCs, peripheral blood mononuclear cells; OD, optical density; DEGs, differentially expressed genes; TRB, TCRβ; ELISA, enzyme-linked immunosorbent assay; HRP, horseradish peroxidase; PBS, phosphate-buffered saline; MDSCs, myeloid-derived suppressor cells; TAMs, tumor-associated macrophages; eTregs, effector Tregs; APCs, antigen-presenting cells; CRD, cysteine-rich domain; EM, effector memory; T1D, type 1 diabetes; NOD, nonobese diabetic; ADCP, antibody-dependent cell-mediated phagocytosis.
