# Supplementary material for: CD137 Agonists Targeting CD137-Mediated Negative Regulation Show Enhanced Antitumor Efficacy in Lung Cancer
Source: Front Immunol. 2022 Feb 7;13:771809. doi: 10.3389/fimmu.2022.771809 (PMC8859117; doi:10.3389/fimmu.2022.771809)
Supplement: Supplementary file 1 [file Table_2.pdf]

Supplementary Table 2 Characteristics of the 82 patients included in the TMA analysis

Characteristic	Number
Age	
<60	33 (40.2%)
≥60	49 (59.8%)
Sex	
Male	71 (86.5%)
Female	11 (13.5%)
Smoker status	
Never	25 (30.5%)
Smoker	57 (69.5%)
Histology	
Adenocarcinoma	35 (42.7%)
Squamous	47 (51.3%)
Stage	
I+II	49 (59.8%)
III+IVA	33 (40.2%)
