# Supplementary material for: CD137 Agonists Targeting CD137-Mediated Negative Regulation Show Enhanced Antitumor Efficacy in Lung Cancer
Source: Front Immunol. 2022 Feb 7;13:771809. doi: 10.3389/fimmu.2022.771809 (PMC8859117; doi:10.3389/fimmu.2022.771809)
Supplement: Supplementary Information — Title: CD137 agonists targeting CD137-mediated negative regulation show enhanced antitumor efficacy in lung cancer. [file Table_1.pdf]

Supplementary Table 1 Correlations between the percentages of Tregs and CD137+ Tregs in the blood and patient characteristics

Variables	Total	Age	% of Tregs	P value	% of CD137+ Tregs	P value
Health controls	34	47.61	7.34	0.1279	3.62	0.0015
Lung cancer patients	29	62.9	8.24		6.05	
Lung cancer patients						
Stage				0.904		0.8524
I+II	12		8.36		6.2	
III+IV	17		8.23		5.95	
Type				0.539		0.4748
Squamous cell	8		8.65		5.375	
Adenocarcinoma	17		8.09		6.67	
Small cell	3		10		4.33	
