# Supplementary figures and images for: CD137 Agonists Targeting CD137-Mediated Negative Regulation Show Enhanced Antitumor Efficacy in Lung Cancer
Source: Front Immunol. 2022 Feb 7;13:771809. doi: 10.3389/fimmu.2022.771809 (PMC8859117; doi:10.3389/fimmu.2022.771809)

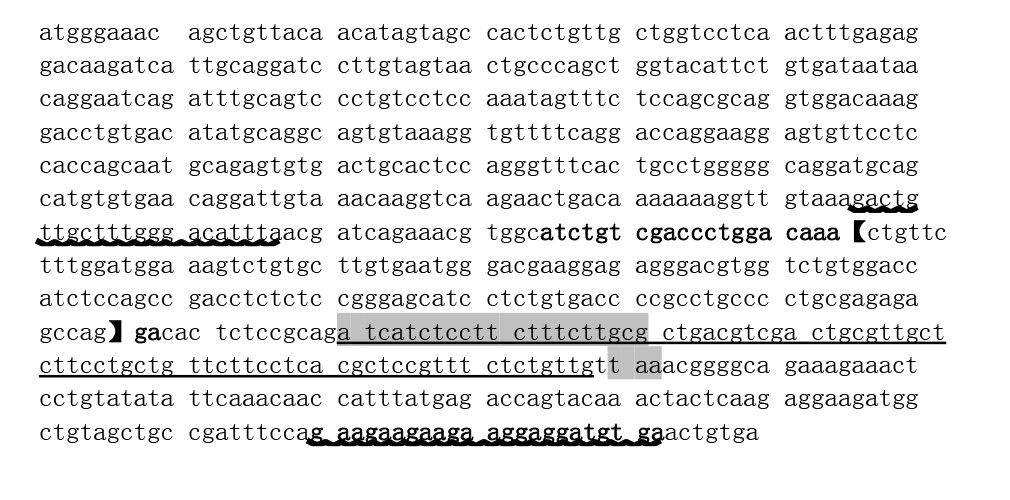

Supplement: Supplementary Figure 1 — mCD137 cDNA sequence and primer sequences. The entire sequence of mCD137 cDNA. The straight underline indicates the transmembrane region. RT–PCR primers (wavy underline) used to amplify sCD137 from activated PBMCs yielded one splice variant that lacked nucleotides 414 to 545 (indicated by brackets). The frame shift results in a stop codon, i.e., the shaded “taa” codon. The sequences of the primers used for ARMS-QPCR are indicated in bold font, and the sequence of the probe is shaded. [file Image_1.jpeg]

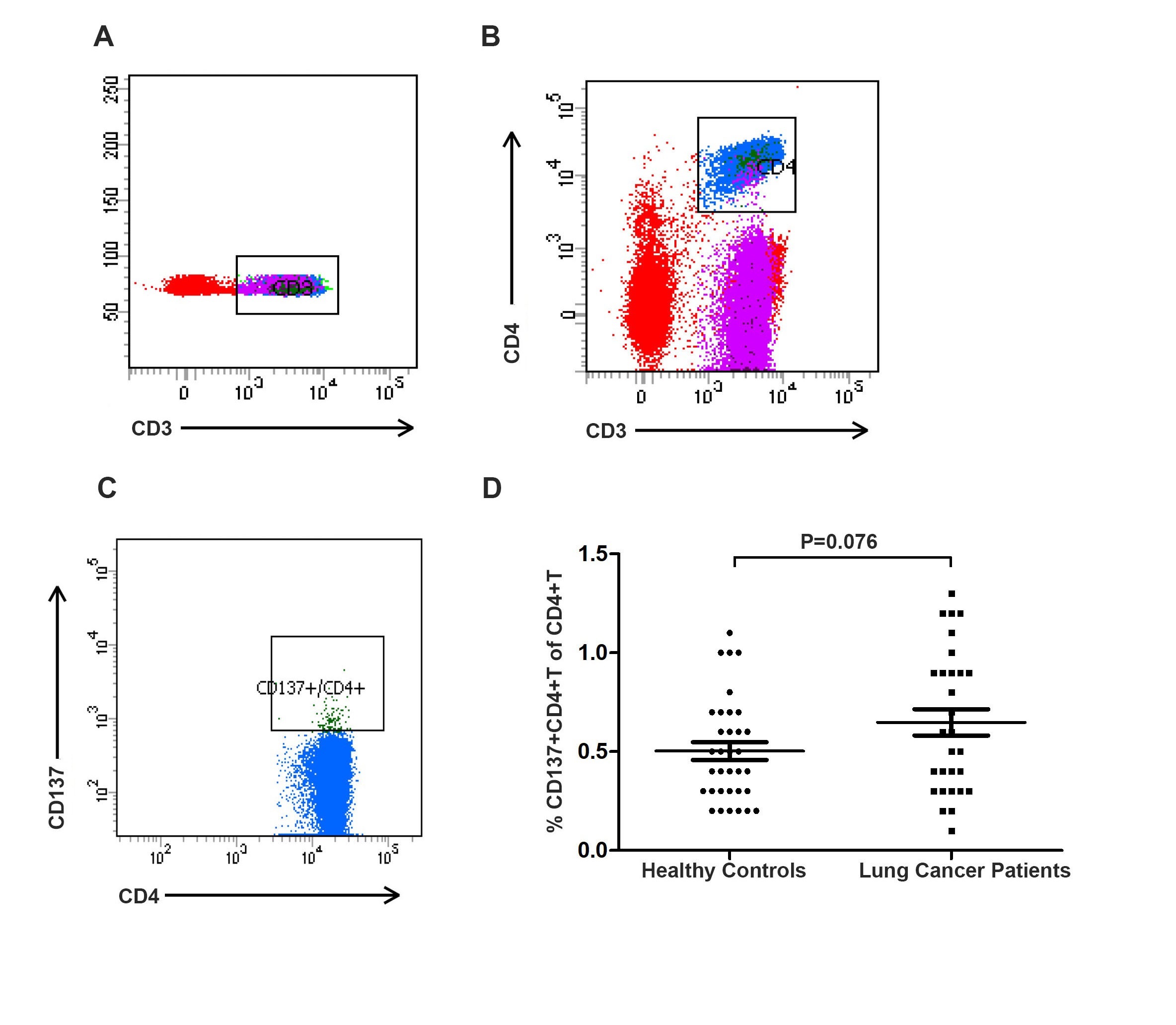

Supplement: Supplementary Figure 2 — Percentage of CD137+CD4+ T cells in the blood. (A) Gated CD3+, (B) CD4+, and (C) CD137+CD4+ T cells. (D) The percentage of CD137+CD4+ T cells in healthy controls and lung cancer patients. The error bars represent the SEMs. [file Image_2.jpeg]

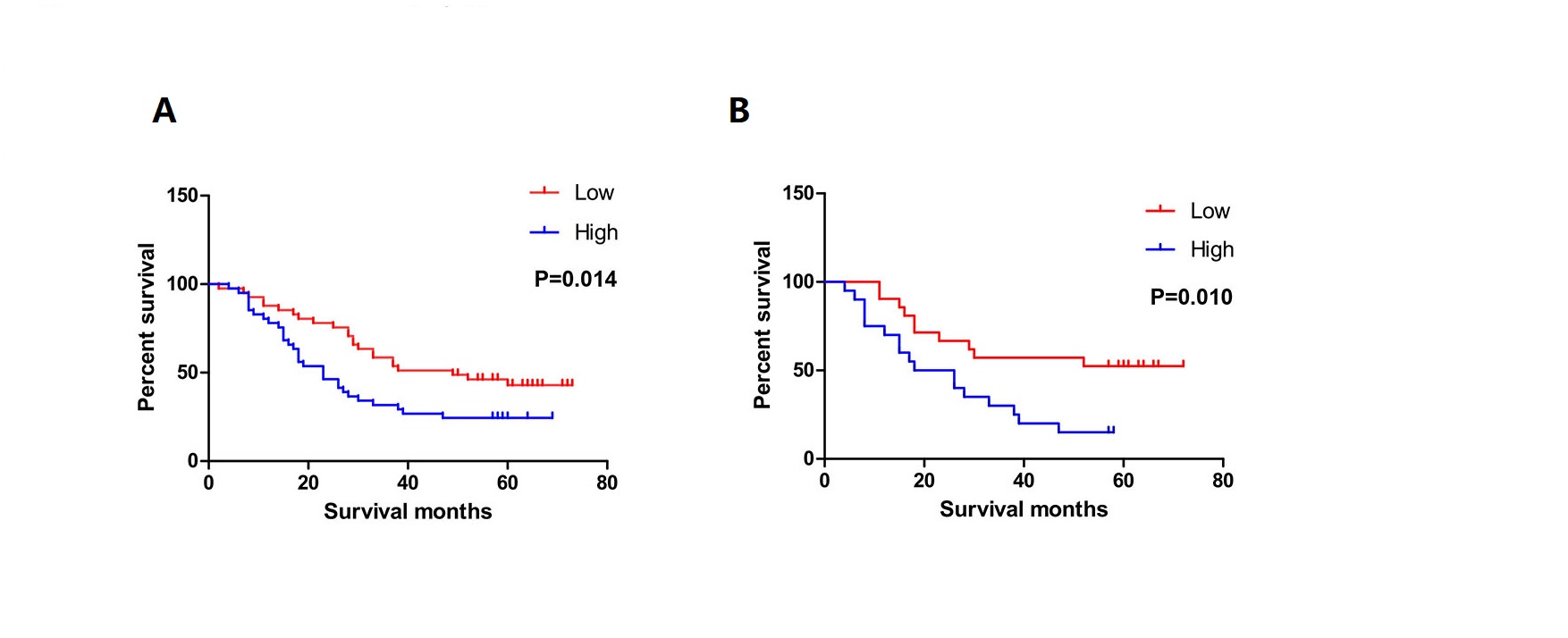

Supplement: Supplementary Figure 3 — Correlation of Treg+ cell density in the tumor microenvironment with OS. Foxp3+ cells in the tumor microenvironment in TMAs from 82 lung cancer patients were detected by multiplexed QIF. (A), Impact of Foxp3+ cell density on patient OS. Dichotomization was based on the median: red line, patients in the low-density group; blue line, patients in the high-density group. Log-rank P values are shown for each graph. (B), Correlation of Foxp3+ cell density with OS in patients with a high number of infiltrating CD137+CD8+ cells in the tumor microenvironment. CD137+CD8+ cell dichotomization was based on the median. [file Image_3.jpeg]

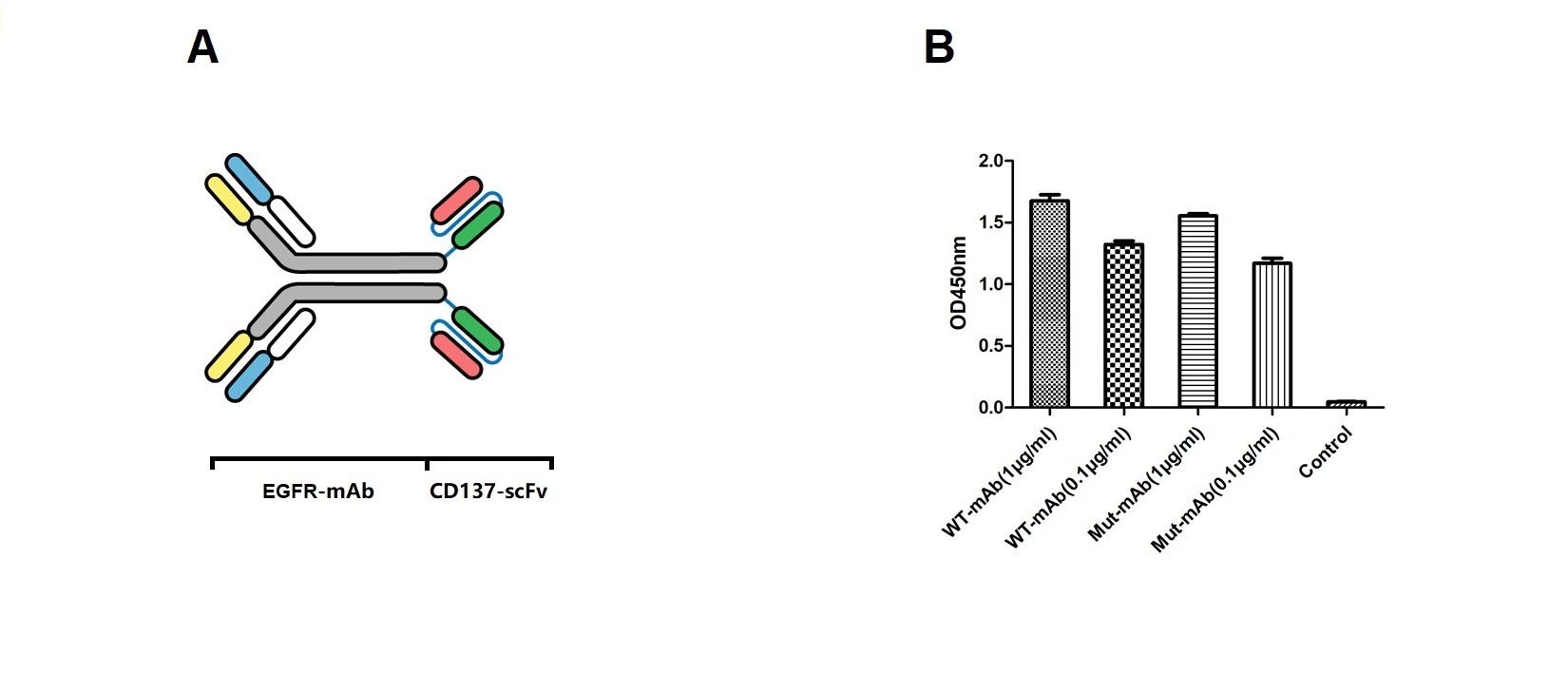

Supplement: Supplementary Figure 4 — Structure and specificity of the therapeutic antibodies. (A), Structural diagram of the therapeutic antibodies used in this study. A wild-type CD137 mAb (Wt-mAb, mIgG2a) and mutant CD137 mAb (Mut-mAb, mIgG2a) with D265A, N297A, L234A, L235A and P329A mutations in FcγR were created via gene synthesis and expressed using a eukaryotic expression system. (B), Binding of therapeutic antibodies to mouse CD137-His. The error bars represent the SEMs. [file Image_4.jpg]

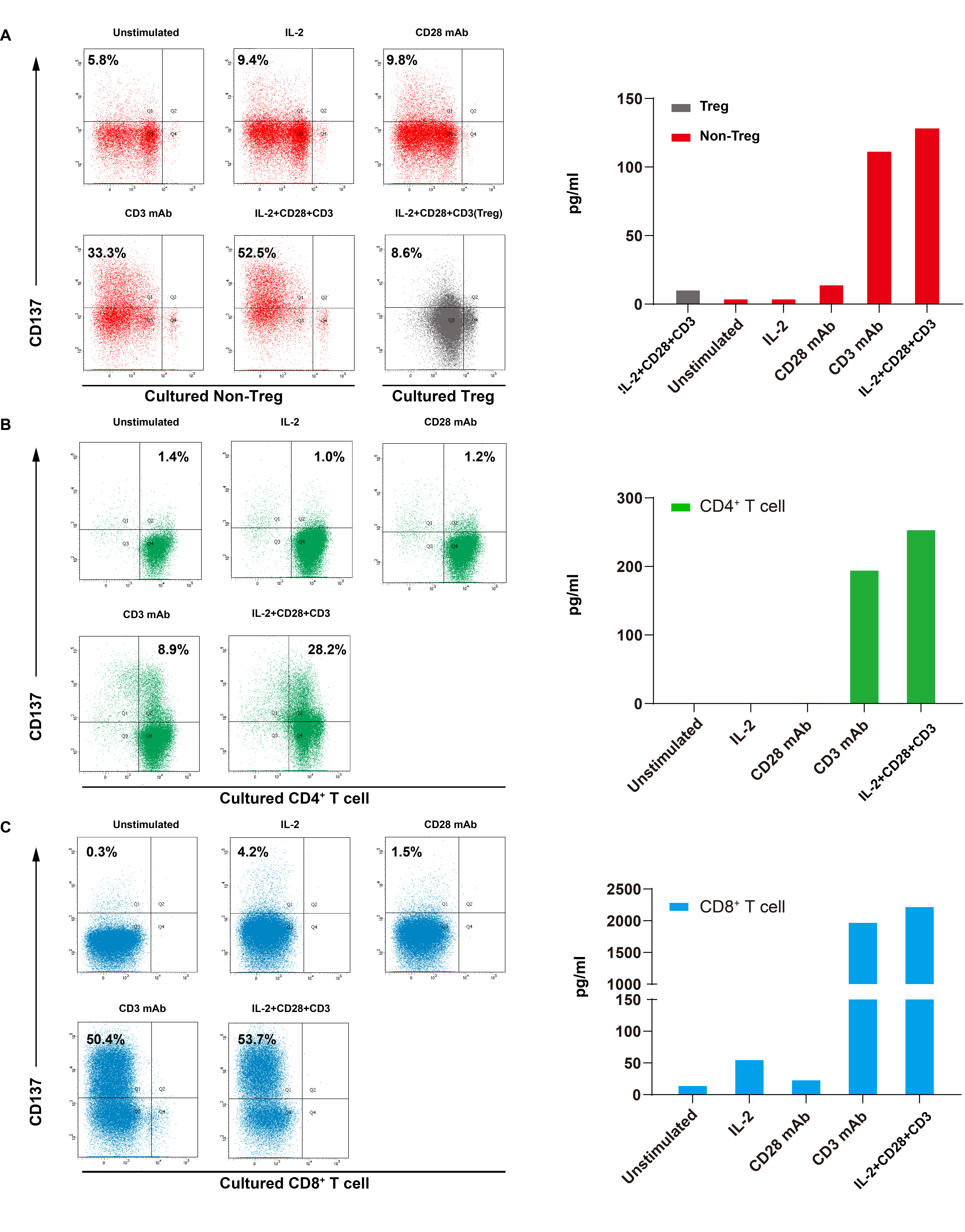

Supplement: Supplementary Figure 5 — Association of mCD137 protein expression with sCD137 protein expression. (A), mCD137 expression and sCD137 levels in cell-free culture supernatant from non-Tregs and Tregs cultured under the indicated cell culture conditions for 3 days. (B), mCD137 expression on CD4+ T subsets grown under the indicated culture conditions and sCD137 levels in the corresponding cell-free culture supernatant. (C), mCD137 expression on CD8+ T subsets grown under the indicated culture conditions and sCD137 levels in the corresponding cell-free culture supernatant. Unstimulated, complete medium only; IL-2, complete medium + IL-2 (100 U/ml); CD28 mAb, complete medium + anti-human CD28 mAb (1 μg/ml); CD3 mAb, complete medium + anti-human CD3 mAb (OKT3, 1 μg/ml); IL-2+CD28+CD3, complete medium + IL-2 + anti-CD28 + anti-CD3; Treg, Treg + complete medium + IL-2 + anti-CD28 + anti-CD3. One representative assay of three experiments is shown. [file Image_5.jpeg]
